# The movie recommendation algorithm based on the TransD model and AIGC empowerment and its application effectiveness analysis

**DOI:** 10.1371/journal.pone.0333607

**Published:** 2025-11-11

**Authors:** Yang Gao, Zhiqun Lin

**Affiliations:** 1 Department of Cinematography, Beijing Film Academy, Beijing, China; 2 Music College of Capital Normal University, Beijing, China; University of Nottingham Ningbo China, CHINA

## Abstract

This study aims to enhance the recommendation system’s capability in addressing cold start issues, semantic understanding, and modeling the diversity of user interests. The study proposes a movie recommendation algorithm framework that integrates Knowledge Graph Embedding via Dynamic Mapping Matrix (TransD) and Artificial Intelligence Generated Content (AIGC)-based generative semantic modeling. This framework is designed to overcome existing challenges in recommendation algorithms, including insufficient user interest representation, inadequate knowledge graph relationship modeling, and limited diversity in recommended content. Traditional recommendation models face three key limitations, including coarse-grained user profiling, reliance on manually generated tags, and inadequate exploitation of structured information. To address these challenges, this study employs the TransD model for dynamic semantic modeling of heterogeneous entities and their complex relationships. Additionally, AIGC technology is employed to automatically extract latent interest dimensions, emotional characteristics, and semantic tags from user reviews, thereby constructing a high-dimensional user interest profile and a content tag completion system. Experiments are conducted using the MovieLens 100K, 1M, and 10M public datasets, with evaluation metrics including Mean Average Precision (MAP), user satisfaction scores, content coverage, click-through rate (CTR), and recommendation trust scores. The results demonstrate that the optimized model achieves hit rates of 0.878, 0.878, and 0.798, and MAP scores of 0.633, 0.637, and 0.574 across the three datasets. The user satisfaction scores are 0.89, 0.88, and 0.87, while the CTR values reach 0.35, 0.33, and 0.34, all of which significantly outperform traditional models. Notably, the proposed approach exhibits superior stability and semantic adaptability, particularly in cold start user scenarios and interest transition contexts. Therefore, this study provides a novel modeling approach that integrates structured and unstructured information for movie recommendation systems. Also, it contributes both theoretically and practically to the research fields of intelligent recommendation systems, knowledge graph embedding, and AIGC-based hybrid modeling.

## Introduction

With the explosive growth of digital media content, users face significantly increased decision-making costs when confronted with massive volumes of film and television resources. As a result, recommendation systems have gradually become one of the core technologies for enhancing user experience and platform revenue [1]. In the domain of movie recommendation in particular, accurately capturing user preferences and providing personalized content has emerged as a key focus of both research and application. However, traditional recommendation algorithms, such as collaborative filtering and content-based methods, still demonstrate limited effectiveness when dealing with cold-start issues, data sparsity, and interest drift [2–4]. In recent years, the knowledge graph (KG) has gained attention as a powerful tool for enhancing the reasoning capabilities of recommendation systems due to its strong semantic modeling capacity. By constructing networks of semantic relationships among entities, KG offers structured contextual information that improves both the interpretability and diversity of recommendations [5]. On this basis, embedding models are widely applied to learn vectorized representations of KG, enabling semantic information to be integrated into recommendation algorithms. Among these models, Knowledge Graph Embedding via Dynamic Mapping Matrix (TransD) introduces a heterogeneous modeling approach for entities and relations. TransD improves representational capacity while maintaining computational efficiency, which makes it particularly suitable for movie recommendation scenarios involving complex relationships [6–8]. Meanwhile, with the rapid advancement of Artificial Intelligence Generated Content (AIGC) technologies, large language models such as Ernie Bot have demonstrated powerful capabilities in text understanding and generation [9]. AIGC can be employed to analyze user reviews, generate interest profiles, and extract content features, thereby providing high-quality supplemental data for recommendation systems. This is especially advantageous in situations with limited user behavioral data or a need for deep semantic understanding.

In summary, integrating the deep semantic modeling capabilities of KG with the generative strengths of AIGC can enable collaborative optimization across key stages of recommendation—such as user modeling, content understanding, and ranking. Concurrently, such integration can evolve recommendation systems from rule-driven to intelligence-driven paradigms. Therefore, this study proposes a movie recommendation framework empowered by the synergy of the TransD embedding model and AIGC technologies. It aims to mitigate the challenges of cold-start and semantic understanding in traditional systems and to achieve breakthroughs in personalized recommendation performance.

## Literature review

Early research on recommendation systems mainly focused on collaborative filtering and content recommendation. Collaborative filtering methods predicted preferences through the similarity among users, but their performance dropped significantly when facing the cold start and data sparsity problems. Torkashvand et al. (2023) pointed out that in the absence of sufficient behavioral data, collaborative filtering was prone to overfitting and struggled to uncover deep-seated preferences [10]. Content-based recommendation focused on item attribute matching; Shahbazi et al. (2025) argued that it was insensitive to user interest drift and likely to cause recommendation redundancy [11]. As recommendation demands diversity, researchers gradually combined recommendation problems with other structured information, which promoted the development of hybrid recommendation systems. Mishra et al. (2024) alleviated some cold-start problems by integrating collaborative filtering with KG, but their method relied on manual feature design and still had limited semantic modeling capabilities [12]. Most of the above methods are mostly static in modeling, respond slowly to changes in user interests, and can hardly adapt to dynamic interaction scenarios. Moreover, cold-start and sparsity problems remain unsolved core challenges, making it urgent to introduce models with stronger generalization capabilities and context understanding abilities. Due to its structured expression ability, the KG becomes an important direction for recommendation systems to enhance semantic understanding. Hussain and Ihsan (2023) constructed an entity relationship network (including actors, directors, styles, etc.) in movie recommendation. They introduced knowledge graph embedding algorithms such as Translating Embeddings for Modeling Multi-relational Data (TransE) and Knowledge Graph Embedding by Translating on Hyperplanes (TransH) to vectorize entities [13]. As a representative subsequent method, the TransD model was proposed by Kapoor et al. (2024) to realize heterogeneous modeling of entities and relationships through dynamic mapping matrices, which improved the representation ability under complex relationships [14]. Compared with early models such as TransE, TransD balanced computational efficiency and expression ability, and was suitable for diversified movie semantic association modeling. However, the above methods are mostly used in basic recommendation tasks and lack integration with user behavior or natural language information. In recent years, the rise of AIGC and large language models inject brand-new semantic modeling capabilities into recommendation systems. Ruan et al. (2025) pointed out that large models could extract emotional tendencies, content preferences, and contextual needs from user reviews, effectively alleviating the limitation of insufficient user historical behavior [15]. Especially in cold-start scenarios, AIGC can automatically generate interest portraits as auxiliary information to enhance recommendation effects. In addition, combining with KG can further strengthen the knowledge understanding ability of recommendation systems. Existing studies mostly treat AIGC and user modeling as parallel inputs, and lack deep modeling of user interest evolution and dynamic semantic alignment mechanisms.

Existing research on recommendation systems has gradually transitioned from single modeling to a complex modeling path that integrates multi-source information. Especially driven by KG, deep learning, and natural language processing technologies, the performance of recommendation systems has been significantly improved. However, a critical analysis of existing literature reveals that current research still has several key gaps. First, semantic modeling and structural modeling have not yet formed effective synergy. Although embedding models such as TransD have advantages in structural expression, they lack sufficient integration of users’ natural language preferences; meanwhile, generative methods such as AIGC are good at processing comment semantics but have weak capabilities in modeling entity relationship structures. Second, current methods are still insufficient in modeling interest evolution. Most methods use static features or time-slice processing, which cannot truly reflect the dynamic change process of user interests. In addition, for cold-start users and extremely sparse data scenarios, although some auxiliary modeling schemes have been proposed, there is no unified modeling paradigm or systematic framework to solve these problems. These gaps limit the adaptability of recommendation systems in complex application environments while providing clear directions for further research.

To address the above issues, this study proposes a hybrid recommendation framework that integrates TransD embedding and AIGC semantic modeling. It aims to bridge the gap between structural expression and semantic understanding, and fundamentally improve the ability of recommendation systems to handle cold-start and dynamic interest problems. The core innovation of this framework lies in the structural-semantic collaborative modeling mechanism. It uses KG to capture entity relationships between users and items. At the same time, it leverages AIGC to mine deep semantic preferences in user reviews, thereby constructing a more comprehensive user profile. To tackle interest drift and data sparsity, the model introduces a dynamic semantic enhancement module; this enables automatic updates of user representations over time and improves the effect of personalized recommendations. Moreover, this study conducts comprehensive experiments on multiple real datasets and uses multi-dimensional indicators to systematically verify the stability and robustness of the proposed model. Concurrently, it provides a clear algorithm reproduction process to enhance the reproducibility and practical reproducibility. This study has certain exploratory value in both theoretical modeling and application practice.

## Research design

### KGE based on TransD

In recommendation systems, KG offers rich capabilities for modeling entity relationships, thereby providing deeper semantic support for recommendations. This is particularly valuable in movie recommendation, where user preferences are often influenced by multiple associated entities, such as actors, directors, genres, countries, and awards [16–18]. As a result, constructing a structured semantic network becomes an essential approach to improving both the accuracy and interpretability of recommendations. TransD is an enhanced method within the family of KGE models, designed to address the limitations of traditional models in representing heterogeneous entities and relationships [19]. The core idea of TransD is to assign each entity and relation two vectors: a representation vector and a projection vector. The projection vectors are then used to dynamically construct mapping matrices that project entities from their original vector space into the relation-specific space, followed by a translation operation in that space. The goal is to ensure that a given triple satisfies the following equation:


$$h_{r}=M_{r}(h),t_{r}=M_{r}(t),h_{r}+r\approx t_{r}$$ (1)

h. t, and r are all elements in the triplet; $M_{r}$ refers to the dynamic mapping matrix calculated by the projection vector. The proposed model can describe the semantic differences between entities and relationships more finely while maintaining computational efficiency.

Based on the movie recommendation scenario, this study constructs a KG that includes the following entities and relationships:

(1) Entity type: Movie, director, actor, genre country, platform, award, etc.;
(2) Relationship type: Directing, acting, belonging, produced from, online in, winning in, and others;
(3) Data source: Public datasets are used in combination with knowledge completion tools (such as a crawler to obtain content).

After the construction, the triplet is input into the TransD model for embedding training. The training objective is to minimize the following scoring functions:


$$f_{r}(h,t)=∥M_{r}(h)+r-M_{r}(t){∥}_{\text{2}}^{\text{2}}$$ (2)

$f_{r}$ represents a scoring function. The error triplet is constructed by a negative sampling mechanism and optimized by margin-based ranking loss to improve the model’s recognition ability for real semantic relationships. After the embedding learning is completed, all entities and relationships are represented as low-dimensional vectors. The recommendation system can leverage these vectors for semantic similarity calculation and interest path expansion [20–22]. For instance, when a user prefers movies featuring specific actor types, the system can identify and recommend similar works by calculating and minimizing the semantic distance of “actor-movie” relationships in the embedding space.

### AIGC-enabled user profile and tag generation

In recommendation systems, user profiling serves as the foundation for achieving personalized recommendations. Traditional user profiles mainly rely on structured behavioral data, which often fail to capture users’ latent interests and emotional preferences in depth [23–25]. With the advancement of AIGC technologies such as large language models, new approaches have emerged for user modeling and content understanding. By leveraging AIGC techniques to interpret and generate user textual data, it becomes possible to construct more refined and dynamic representations of user interests. This study employs AIGC-based methods to semantically analyze and generatively model user interests, stylistic preferences, and emotional tendencies based on users’ textual behaviors on the platform. The specific steps are exhibited in Table 1.

**Table 1:** Generation steps of user profiles.

Step	Description
Data collection and preprocessing	The user’s textual data on the platform are collected, with noise information removed, and undergo preprocessing operations including text segmentation, stop word removal, and emotion normalization.
Semantic analysis and topic modeling	Using the large language model combined with topic modeling technology, this study analyzes the core themes in user comments and extracts user concerns (such as “complex plot”, “compact rhythm”, “good actor performance”, etc.).
Emotional tendency recognition and style preference generation	The emotion recognition model is used to extract the user’s emotion and attitude, and the following user preference dimensions are generated combined with semantic tags: Preference types (such as drama, suspense, and art) Emotional tendencies (such as positive, neutral, sad, and excited) Content preference (such as plot reversal, visual effects, and character growth)
Construction of multi-dimensional user profile vector	The above analysis results are structured into vector form as one of the inputs of the recommendation system, and participate in the recommendation process together with the KG information embedded in TransD.

Movie content tagging is traditionally dependent on manual annotation or basic keyword extraction, which often results in issues such as limited tag coverage and semantic ambiguity. To address these limitations, this study introduces the content generation capabilities of AIGC to produce rich, specific, and semantically clear content tags. The tagging process mainly includes:

(1) Plot tag generation: Given movie synopses or review summaries as input, the model outputs concise plot descriptors (e.g., “family ethical conflict”, “coming-of-age journey,” “time-loop setting”);
(2) Style tag induction: By analyzing common expressions in user reviews (e.g., “dark humor,” “visual spectacle,” “fast-paced”), the model automatically classifies descriptions into interpretable stylistic dimensions;
(3) Emotional tag matching: Through semantic similarity analysis, the system maps users’ emotional expressions to movie content, enabling emotion-driven plot recommendations.

Through this mechanism, the system semantically enriches existing content and generates new tag dimensions for long-tail items, thus enhancing its ability to handle less popular and newly released movies.

### Integration and optimization of recommendation algorithms

**Fig 1:**
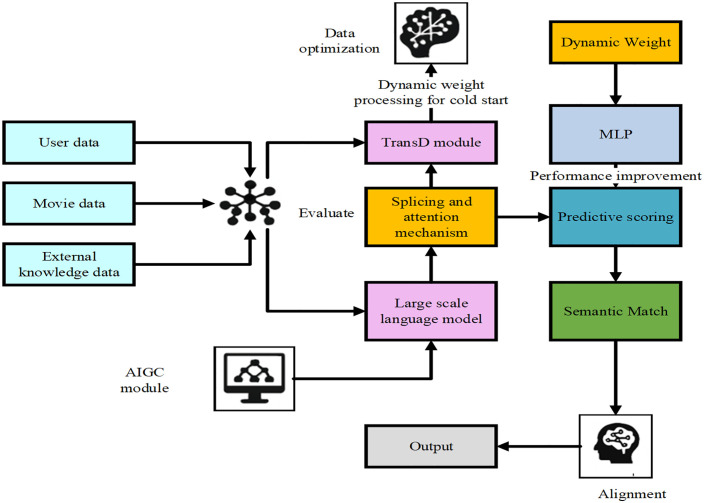
Fusion framework.

In recommendation systems, the core objective of algorithms is to accurately match user interests with item features to maximize the effectiveness of personalized recommendations. The previous two sections provide data support and representation strategies from two perspectives: structured semantics and generative semantics. This section introduces how to integrate these two types of semantic information into a unified recommendation algorithm framework. It aims to optimize recommendation performance, enhance system robustness, and improve adaptability. The recommendation algorithm architecture proposed in this study comprises three primary layers:

(1) The underlying semantic modeling layer: This layer focuses on constructing fundamental representations for users and items, based on both structured and unstructured input data. The structured part leverages KG triples and employs the TransD embedding model to learn representations of various entities such as users, movies, directors, and genres. By introducing dynamic mapping matrices, TransD projects heterogeneous entities into relation-specific spaces, thus enhancing the model’s generalization ability in multi-relational semantic contexts. The unstructured part utilizes large language models to generate semantic tags and perform vector encoding. By analyzing textual data such as user reviews and rating comments, the system extracts interest topics, emotional features, and content inclinations to construct user-semantic interest vectors and item semantic tag vectors, forming the semantic foundation for subsequent recommendation tasks.
(2) The intermediate feature fusion layer: This layer plays a critical role in unifying structured and unstructured information. It first concatenates users’ and items’ structural and semantic vectors to form initial fused input representations. A cross-attention mechanism is then introduced to guide the model in learning alignment patterns between structural and semantic features, allowing it to dynamically adjust feature weights based on semantic discrepancies. For users or items with rich entity information but sparse reviews, structural features are weighted more heavily; whereas for cold-start users with limited behavior data, semantic features offer valuable compensation. Additionally, to enhance the contextual awareness of structural embeddings, a Graph Attention Network (GAT) may be employed to aggregate TransD outputs, producing more precise node representations that further support effective feature fusion.
(3) The top recommendation decision layer: This layer is responsible for behavior prediction and generating recommendation outputs based on the fused representations. The integrated vectors are fed into a Multilayer Perceptron (MLP), which learns a nonlinear scoring function to predict user preferences for specific items. During the ranking stage, the system generates recommendation lists based on the predicted scores. Meanwhile, it applies auxiliary optimization by considering factors such as the user’s current interest state, content diversity, and novelty. In cold-start scenarios, the system falls back to using AIGC-generated semantic vectors in place of structural vectors and performs recommendations based on semantic similarity, effectively mitigating the information sparsity problem. The ultimate goal of this layer is to produce personalized, interpretable, and exploratory recommendation outputs, forming the behavioral logic endpoint of the complete recommendation system. The overall architecture is illustrated in Fig 1.

Through calculation, the final recommendation score can be expressed as equation (3):


$$\text{Score}(u,i)=\sigma \left({\text{W}}_{\text{1}}[{\text{e}}_{u}∥{\text{e}}_{i}]+{\text{W}}_{\text{2}}[{\text{g}}_{u}∥{\text{g}}_{i}]+b\right)$$ (3)

Score refers to the recommendation score; u is the user; i represents a movie project; ${\text{W}}_{\text{1}}$ and ${\text{W}}_{\text{2}}$ are weight parameters; b denotes the offset term; ${\text{e}}_{u}$ and ${\text{e}}_{i}$ stand for embedding vectors; ${\text{g}}_{u}$ and ${\text{g}}_{i}$ are content tags; σ means the activation function. To further improve the accuracy of semantic matching, a cross-attention mechanism can be introduced to guide users to generate alignment matching between interest and movie tags.

In short, the proposed hybrid algorithm framework achieves an organic integration of the structural representation capabilities of the TransD and the cognitive understanding abilities of AIGC-based semantic generation. This integration enhances the accuracy and personalization of the recommendation system while improving its adaptability to cold-start users and the interpretability of recommendations. The framework is highly extensible and can be flexibly embedded into various recommendation scenarios, making it suitable for most data platforms that contain textual content and relational networks.

### The design of experiments

The dataset used in this study is the MovieLens dataset, which is collected by the GroupLens research team at the University of Minnesota and contains user ratings of movies. The dataset is available in multiple versions to support research at different scales. It can be downloaded from: https://tianchi.aliyun.com/dataset/146674?utm_source=chatgpt.com. The version of the dataset selected for the experiments in this study is as follows:

(1) MovieLens 100K: It includes 100000 scoring records of 1682 movies by 943 users.
(2) MovieLens 1M: It encompasses 1000209 scoring records of 4000 movies by 6000 users.
(3) MovieLens 10M: It contains 100000054 scoring records of 69878 users on 10000 movies.

Some codes in the model construction are as follows:

**   **class TransD(nn.Module):
  **     **def __init__(self, num_entities, num_relations, embedding_dim):
  **     **super(TransD, self).__init__()
  **     **self.entity_embedding = nn.Embedding(num_entities, embedding_dim)
  **     **self.relation_embedding = nn.Embedding(num_relations, embedding_dim)
  **     **self.entity_proj = nn.Embedding(num_entities, embedding_dim)
  **     **self.relation_proj = nn.Embedding(num_relations, embedding_dim)

To ensure the stability and repeatability of the experiment, the hardware configuration is planned in detail:

(1) Central Processing Unit (CPU) model: Intel Core i9-12900K
(2) Graphic Processing Unit (GPU) model: NVIDIA RTX 3090 (24GB video memory)
(3) Memory capacity: 64GB DDR5
(4) Motherboard model: ASUS ROG STRIX Z690
(5) Operating system version: Ubuntu 20.04 LTS (64-bit)

Parameter settings are outlined in Table 2:

**Table 2:** Parameter settings.

Name of parameter	Parameter values
Vector embedding dimension	100
Interval parameter in the loss function	1.0
Learning rate	0.001
Optimizer type	Adam
Number of training samples per batch	512
Number of training epochs	100
Dropout rate	0.3
Batch size	256
Loss function type	BPR Loss
Recommended list length	Top-10
Negative sampling ratio	5:1
Distance measurement method (L1 or L2)	L2

This study selects the following models for comparison: Knowledge Graph Induction Network (KGIN), Knowledge Graph Contrastive Learning (KGCL), Self-Attentive Sequential Recommendation (SASRec), Self-Supervised Learning for Sequential Recommendation (S3-Rec), and Deep Interest Network (DIN). These five models represent diverse research directions, including graph neural networks, sequential modeling, knowledge-enhanced recommendation, and deep interest modeling, making them suitable baselines for comparison with the proposed hybrid model.

## Algorithm evaluation

### Performance analysis

**Fig 2:**
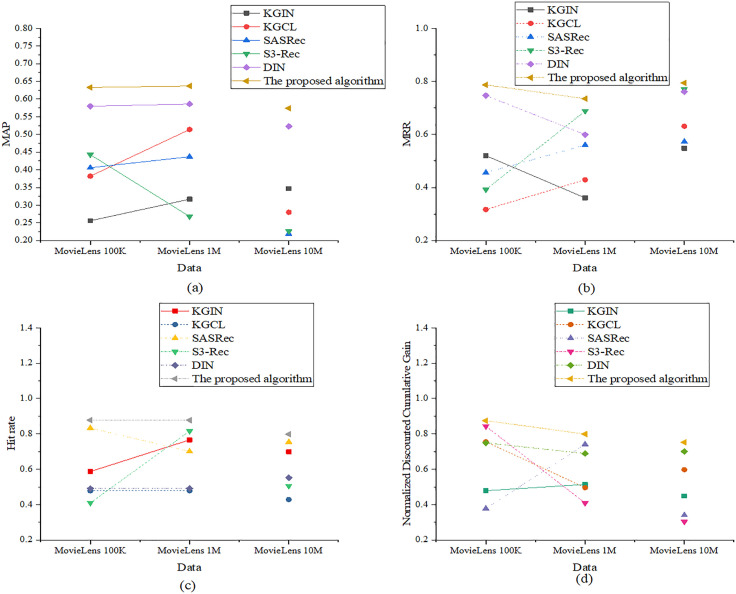
Recommendation performance evaluation ((a): MAP; (b): MRR; (c): Hit Rate; (d): nDCG).

**Fig 3:**
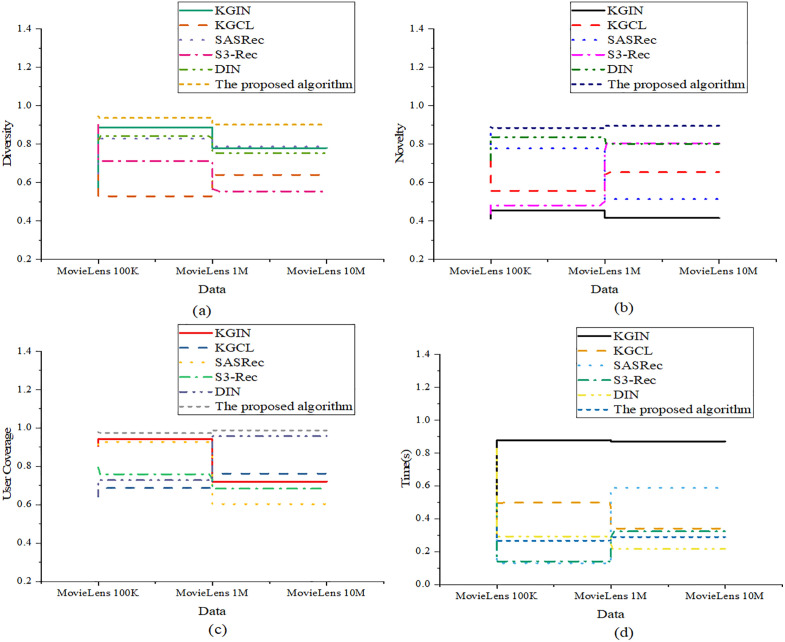
Evaluation of system performance and user experience ((a): Recommendation diversity; (b): Recommendation novelty; (c): User coverage; (d): Average response time).

In the performance comparison experiments, this study evaluates the models from two dimensions: recommendation effectiveness and system performance/user experience. Each dimension includes four core metrics to ensure a comprehensive and fair assessment. The evaluation metrics for predictive performance are Mean Average Precision (MAP), Mean Reciprocal Rank (MRR), Hit Rate, and Normalized Discounted Cumulative Gain (nDCG). The results of the recommendation effectiveness evaluation are presented in Fig 2:

Fig 2 indicates that in terms of hit rate, the optimized model has high values of 0.878, 0.878, and 0.798 on the three datasets, respectively. In comparison, while SASRec reaches 0.833 on MovieLens 100K, its performance drops significantly on MovieLens 10M. For the MAP metric, the optimized model records 0.633, 0.637, and 0.574, outperforming DIN’s 0.586 on MovieLens 1M. Regarding MRR, the optimized model achieves 0.787, 0.735, and 0.794 across the three datasets, while S3-Rec reaches a comparable 0.770 only on MovieLens 10M. In the more comprehensive ranking quality metric, nDCG, the optimized model yields results of 0.875, 0.799, and 0.752, significantly surpassing S3-Rec’s 0.409 on MovieLens 1M. For the system performance and user experience dimension, the metrics encompass recommendation diversity, novelty, user coverage, and average response time. The corresponding evaluation results are revealed in Fig 3:

In Fig 3, the optimized model exhibits superior performance in recommendation quality metrics, particularly in diversity and novelty. Across all three datasets, the model achieves diversity scores of 0.945, 0.937, and 0.902, along with novelty scores of 0.889, 0.884, and 0.895, demonstrating consistent excellence in both dimensions. In comparison, S3-Rec achieves a diversity score of 0.899 on MovieLens 100K but only 0.554 on MovieLens 10M. Regarding user coverage, the optimized model reaches 0.978, 0.974, and 0.987 on the three datasets, respectively, whereas KGCL only achieves 0.642 on MovieLens 100K, indicating a notable gap. Finally, in terms of system response efficiency, the optimized model maintains low average response times of 0.345s, 0.266s, and 0.289s, outperforming DIN, which records 0.827s on MovieLens 100K.

### Comparative analysis of simulation results for movie recommendation

**Fig 4:**
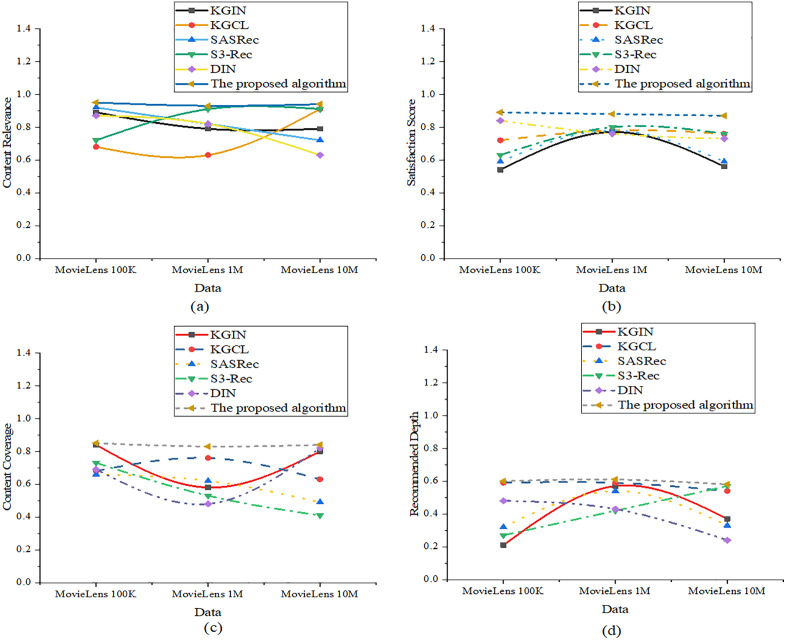
Evaluation of fine-grained recommendation quality ((a): Content relevance; (b): Predicted user satisfaction scores; (c): Content coverage; (d): Recommendation depth).

**Fig 5:**
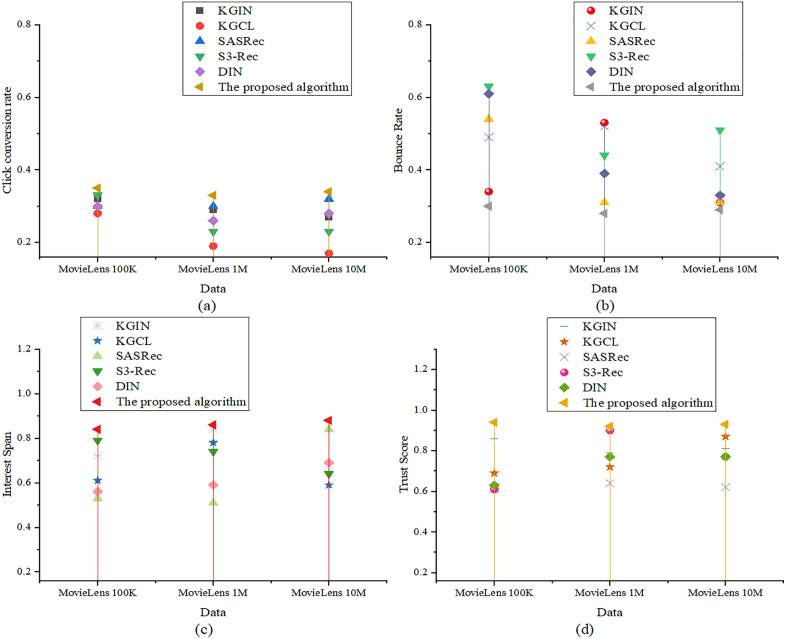
Evaluation of user experience and behavior ((a): CTR; (b): Bounce rate; (c): Interest span; (d): Recommendation trust score).

The experiment also evaluates performance from two dimensions: fine-grained recommendation quality and user experience/behavior. The metrics under fine-grained recommendation quality include content relevance, predicted user satisfaction scores, content coverage, and recommendation depth. The results are suggested in Fig 4:

In Fig 4, the optimized model attains content relevance scores of 0.95, 0.93, and 0.94 on the three datasets, respectively, significantly outperforming KGCL’s 0.63 on MovieLens 1M. This indicates the model’s superior ability to identify users’ thematic interests and match them with relevant content. For predicted user satisfaction scores, the optimized model achieves 0.89, 0.88, and 0.87, demonstrating consistently high recommendation appeal, and surpassing DIN’s 0.76 on MovieLens 1M. Regarding content coverage, the optimized model scores 0.85, 0.83, and 0.84, effectively covering users’ multidimensional preferences and avoiding content homogeneity. In contrast, S3-Rec exhibits only 0.41 on MovieLens 10M, indicating a tendency toward repetitive content. For recommendation depth, the optimized model records values of 0.60, 0.61, and 0.58 across the datasets, which are generally superior to SASRec’s 0.33 on MovieLens 10M. This reflects a more balanced distribution from mainstream to niche content. The user experience and behavior dimension includes the following metrics: click-through rate (CTR), bounce rate, interest span, and recommendation trust score. The corresponding results are depicted in Fig 5:

Fig 5 illustrates that concerning CTR, the optimized model achieves scores of 0.35, 0.33, and 0.34, all higher than KGCL’s 0.17 on MovieLens 10M. This demonstrates that the recommended content is highly appealing to users. Regarding bounce rate, the optimized model records 0.30, 0.28, and 0.29, which are significantly lower than the 0.63 observed for S3-Rec on MovieLens 100K, suggesting that the recommendations better align with user expectations. In terms of interest span, the optimized model scores 0.84, 0.86, and 0.88, demonstrating a strong ability to distribute content across diverse user interest areas, outperforming SASRec’s 0.51 on MovieLens 1M. Finally, for the recommendation trust score, the optimized model reaches 0.94, 0.92, and 0.93, considerably higher than SASRec’s 0.62 on MovieLens 10M, reflecting the superior trustworthiness of the recommendation system.

## Discussion

The performance comparison results show that the integrated recommendation model proposed in this study outperforms existing mainstream models across multiple dimensions, including overall recommendation quality, ranking performance, system efficiency, and user experience. This advantage primarily stems from the model’s innovations in semantic modeling and information fusion strategies. On one hand, the TransD embedding model enhances the semantic representation capabilities of the structured KG, improving the model’s ability to perceive user interest paths and item attributes. On the other hand, AIGC-based user profiles and content tags effectively compensate for the limitations of traditional models in handling cold-start scenarios and semantic understanding, remarkably improving the recommendation system’s adaptability and generalization capability. Moreover, the proposed model performs excellently in improving recommendation diversity and novelty, illustrating its ability to balance personalized user exploration with content discovery, thus mitigating the “information cocoon” effect. While ensuring high recommendation quality, the proposed model maintains efficient runtime performance, making it suitable for practical deployment. Although some traditional models perform comparably in individual metrics, the overall superiority in stability and comprehensive indicators validates the proposed model’s rationality and effectiveness. The simulation-based comparative analysis demonstrates that the optimized model consistently achieves superior performance across multiple dimensions, indicating its strong capability in enhancing both overall recommendation quality and user experience. This performance stems from the model’s dual design in user modeling and semantic tag integration. The structured KG provides high-quality entity relationship information, while AIGC-based user interest tags and content descriptions enhance the depth and breadth of semantic matching. Moreover, improvements in content hierarchy, diversity, and coverage help the system better balance between “precision” and “discovery,” thus enhancing the overall recommendation experience. From the perspective of user behavior, the optimized model effectively reduces bounce rates while increasing CTR and trust scores. This suggests that the system’s recommendations better align with user needs and are more engaging and trustworthy.

Zhang et al. (2025) proposed an attention mechanism-based user-item interaction model, emphasizing dynamic modeling of behavioral sequences, but exhibiting limited capabilities in modeling unstructured text and cold-start users [26]. Compared with Zhang et al. (2025)‘s model, the proposed model demonstrates stronger semantic robustness and structural comprehension ability in addressing interest migration issues. Moreover, this study generates user interest tags through AIGC technology and combines them with structured KG, maintaining high recommendation accuracy and user coverage in cold-start scenarios. The user coverage on the MovieLens 100K dataset reaches 0.978, significantly higher than the performance of their model. Foo et al. (2025) argued that AIGC could generate movie summaries and enhance content understanding, which mainly focused on content-side enhancement but lacked structured modeling of user behavior [27]. Compared with their research, this study further achieves deep integration of structural and semantic information. The model proposed in this study introduces TransD to model multiple semantic paths between users and entities, combining semantic interest tags for synergistic enhancement. This improves content relevance (reaching 0.93 on MovieLens 1M). It also achieves higher performance in subjective experience metrics such as recommendation trust (0.93 on MovieLens 10M), verifying the effectiveness of the structural-semantic collaborative mechanism.

## Conclusions

With the continuous evolution of big data and AI technologies, recommendation systems are gradually moving towards higher levels of intelligence and personalization. To address the limitations of traditional recommendation algorithms in cold-start scenarios, interest understanding, and semantic modeling, this study proposes a movie recommendation algorithm that integrates TransD with AIGC mechanisms. Built upon multi-source heterogeneous data, the structured component of the model employs the TransD framework to meticulously model user-entity-relationship interactions. In contrast, the unstructured component leverages large language models to process text data such as user reviews, automatically generating semantic interest tags to achieve a synergistic representation of user profiles and item features. Experimental results demonstrate significant advantages of the optimized model across multiple recommendation metrics. For instance, on the MovieLens 100K dataset, it achieves a hit rate of 0.878 and an nDCG of 0.875, both outperforming traditional graph neural network models. Additionally, regarding user experience, the proposed model excels in both content novelty and user coverage, validating the positive impact of structural-semantic fusion on enhancing personalized recommendation effectiveness. In system response efficiency, the average response time is controlled within 0.3 seconds, indicating good deployment adaptability and real-time service capabilities. Despite these advancements, the proposed model still has certain limitations. For example, the quality of tags generated by AIGC depends on the expressive power of pre-trained models, potentially leading to insufficient semantic generalization. Moreover, in large-scale data environments, the computational overhead of TransD modeling and semantic reasoning requires further optimization. Future research could incorporate multimodal data to enrich the dimensionality of semantic understanding and explore joint optimization methods for KG and generative models to further improve the model’s expressive precision and practical utility.

## Supporting information

S1 Data The data in the figures in the manuscript can be found in the supporting information “Data in Figures.zip”. (ZIP)
