# Supplementary material for: The movie recommendation algorithm based on the TransD model and AIGC empowerment and its application effectiveness analysis
Source: PLoS One. 2025 Nov 11;20(11):e0333607. doi: 10.1371/journal.pone.0333607 (PMC12604752; doi:10.1371/journal.pone.0333607)
Supplement: S1 Data — (ZIP) [file pone.0333607.s001.zip › 数据包/Code Description.docx]

**1. TransD Knowledge Graph Embedding Module**

1.1 Overview

This section introduces a class named TransD, which inherits from nn.Module, the base class for all neural network models in PyTorch. This inheritance indicates that the module functions as a trainable sub-network.

1.2 Initialization

The __init__() function initializes the module with three parameters: the number of entities, the number of relations, and the embedding dimension. The model initializes two distinct embedding vectors for both entities and relations: a representation vector (entity_embedding, relation_embedding) and a projection vector (entity_proj, relation_proj). This design is the core of TransD, effectively addressing the issue of entity-relation dimensional mismatch and enhancing adaptability to heterogeneous knowledge graph structures.

1.3 Projection Mechanism

The _project() function implements the dynamic projection operation proposed in TransD. It projects entity vectors into the corresponding relation space using their respective projection vectors, thereby enabling relation-specific transformations. This mechanism enhances the semantic representation of entities and improves the accuracy of triple modeling.

1.4 Forward Computation

The forward() method takes as input the indices of the head entity (h), relation (r), and tail entity (t). It retrieves the corresponding embeddings via lookup operations, performs the aforementioned projection and transformation processes, and ultimately computes a score using the Euclidean distance (L2 norm). This score measures the semantic plausibility of a given triple, where a lower score indicates a higher degree of semantic coherence.

1.5 Summary

Overall, this module provides a lightweight modeling mechanism for knowledge graph embedding learning. The generated structural-semantic vectors can be further integrated into downstream tasks to characterize the positional and relational features of users and items within the knowledge graph.

**2. AIGC Semantic Vector Generation Module**

2.1 Overview

AIGCSemanticEncoder is an independent class designed to invoke a pretrained language model for extracting textual semantic vectors from user reviews or movie descriptions.

2.2 Initialization

The initialization function loads a sentence encoding model provided by Hugging Face Transformers (default: all-MiniLM-L6-v2). This model is a lightweight variant of BERT, optimized for efficient semantic vectorization of short texts such as reviews and summaries.

2.3 Encoding Process

The encode() method takes a set of textual inputs, which are first tokenized and then passed through the Transformer model for forward propagation. The final semantic representation of each text is obtained by averaging the last-layer hidden states of the model’s output.

2.4 Advantages and Applications

This model can be directly utilized without additional training, effectively addressing the issue of sparse semantic representations. Furthermore, it exhibits strong transferability, making it particularly suitable for cold-start user profiling and content tag generation.

**3. Cross-Attention Feature Fusion Module**

3.1 Overview

The CrossAttentionFusion module is a neural network substructure designed to integrate structural vectors and semantic vectors, leveraging PyTorch’s multi-head attention mechanism (nn.MultiheadAttention) as its core component.

3.2 Design Objective

The primary goal of this module is to bridge the information gap between structural representations and semantic representations. By employing a cross-attention mechanism, it explicitly aligns these two types of information, thereby enhancing the semantic precision of the fused representation.

3.3 Forward Computation

In the forward() method, the structural and semantic vectors of users or items are first expanded into two-dimensional matrices before being processed by the multi-head attention module. The resulting interaction features are then transformed into a unified-dimensional fused vector through a linear layer.

3.4 Adaptive Information Weighting

This module exhibits dynamic perception capabilities, allowing it to automatically adjust the weighting between structural and semantic information based on semantic similarity. This adaptability makes it particularly well-suited for scenarios where user data is incomplete or content tags are insufficient.

**4. HybridRecommender Main Model Module**

4.1 Overview

HybridRecommender serves as the primary model of the recommendation system, integrating the three aforementioned modules while additionally incorporating a rating prediction network.

4.2 Model Initialization

During initialization, the model defines embedding tables for users and items and instantiates the TransD model, AIGC semantic encoder, and cross-attention fusion module.

4.3 Rating Prediction Network

The rating prediction network consists of a three-layer fully connected neural network (MLP), which takes as input the fused user-item vectors and applies ReLU activation and linear transformations to generate the final predicted rating score.

4.4 Forward Computation

In the forward() method:

Structural embeddings for users and items are retrieved based on their respective indices.

The semantic encoding module is invoked to vectorize user and item text data.

The cross-attention fusion module processes both structural and semantic vectors to generate fused representations for users and items.

The resulting fused vectors are concatenated and passed into the rating predictor, which outputs the predicted user-item interaction score.

4.5 Key Features

This module supports end-to-end training and is capable of handling cold-start scenarios, semantic reasoning, and relation modeling. As the core component of the system, it enables diverse feature representations, enhancing the overall recommendation performance.

**5. Cold-Start Handling Function**

5.1 Overview

The handle_cold_start() function is designed to address the cold-start problem, which arises when users or items lack historical interaction data, making collaborative filtering inapplicable for recommendations.

5.2 Methodology

The function directly invokes the semantic encoder to vectorize user and item textual descriptions. It then computes the cosine similarity between these vectors to measure the semantic alignment between users and items, which is used as the predicted rating score.

5.3 Application and Advantages

This strategy enables "recommendation without historical data", making it particularly suitable for new users, new items, or new content during the cold-start phase. By leveraging semantic matching, it ensures the stability and broad coverage of the recommendation system.

**6. Example Execution**

6.1 Model Initialization

In the __main__ section, a well-parameterized recommender instance is created, specifying key settings such as the number of users, items, entities, and relations.

6.2 Input Simulation

Three sets of user and item indices are defined, along with their corresponding natural language reviews or descriptions, simulating real-world recommendation system inputs awaiting rating predictions.

6.3 Model Execution

The model is invoked to perform a forward prediction, and the predicted rating scores are printed. Additionally, the cold-start handling function is executed to generate fallback predictions, outputting semantic similarity-based scores for cold-start scenarios.

6.4 Validation and Demonstration

This example verifies the integration of different model components and showcases the operational workflow of the recommender system in both data-rich environments and cold-start scenarios, demonstrating its adaptability and effectiveness.
