# Supplementary material for: The movie recommendation algorithm based on the TransD model and AIGC empowerment and its application effectiveness analysis
Source: PLoS One. 2025 Nov 11;20(11):e0333607. doi: 10.1371/journal.pone.0333607 (PMC12604752; doi:10.1371/journal.pone.0333607)
Supplement: S1 Data — (ZIP) [file pone.0333607.s001.zip › 数据包/Dataset Description.docx]

**MovieLens 100K Dataset**

MovieLens 100K is a benchmark dataset released by the GroupLens Research Lab at the University of Minnesota, USA. It is widely used in academic research on collaborative filtering, personalized recommendation, and knowledge graph-enhanced recommendation systems. Due to its well-structured format and moderate scale, this dataset facilitates rapid prototyping and algorithm validation, making it particularly suitable for evaluating model effectiveness in small- to medium-scale scenarios.

**1. Dataset Overview**

The MovieLens 100K dataset consists of a total of **100,000** rating records, involving **943** users and **1,682** movies. With its broad coverage, the dataset effectively captures the diversity of user behaviors and preference variations, making it an essential resource for evaluating recommendation models.

**2. Dataset Structure**

The MovieLens 100K dataset primarily consists of the following four data files:

- **u.data:** The core rating file, recording user ratings for various movies.
- **u.item:** The movie information file, containing metadata such as movie ID, title, release date, and genre.
- **u.user:** The user information file, providing basic user attributes (e.g., age, gender, occupation, and ZIP code).
- **u.genre:** The movie genre file, specifying the genre classification of each movie (e.g., action, romance, science fiction).

**3. Rating Data Format**

The rating data is stored in the u.data file, using tab-separated values with the following format:

user_id item_id rating timestamp

- user_id: User ID, ranging from 1 to 943.
- item_id: Movie ID, ranging from 1 to 1682.
- rating: User-assigned rating for the movie, represented as an integer from 1 to 5.
- timestamp: A Unix-format timestamp indicating the exact time (in seconds) when the rating was given.

**4. User Attribute Information**

The u.user file contains the following attributes for each user:

- Age (age): Ranges from 7 to 73 years old.
- Gender (gender): The male-to-female ratio is approximately 72:28.
- Occupation (occupation): Includes 21 different types of professions, such as student, educator, engineer, writer, and doctor.
- ZIP Code (zip code): Can be used for geographical distribution analysis (optional feature).

These attributes facilitate user profiling and interest-based segmentation for recommendation modeling.

**5. Movie Metadata**

The u.item file provides detailed movie attribute information, including:

- Movie ID & Title: The unique identifier and title of each movie.
- Release Date: The date when the movie was released.
- Movie Genre: The genre of the movie, with a total of 19 categories, such as Action, Comedy, Romance, Sci-Fi, etc.
- These attributes can be used to construct item attribute nodes in a knowledge graph or to build a movie genre feature matrix for further analysis and modeling.

**6. Data Distribution Characteristics**

- Moderate Data Sparsity: On average, each user has rated approximately 106 movies, and each movie has been rated about 59 times.
- Significant Long-Tail Phenomenon: Some popular movies have received many ratings, while a large number of less popular movies have relatively few ratings.
- Stable Rating Preferences: The distribution of ratings is left-skewed, with ratings between 3 to 5 being dominant, indicating that many users tend to show a neutral rating bias.
- Non-Uniform User Behavior: Some users exhibit significantly higher activity levels than average, engaging in multiple interactions.

**7. Data Application Advantages**

- Model Validation: The dataset can be used to validate the effectiveness of collaborative filtering, content-based recommendation, hybrid recommendation, and deep learning models.
- Enriched Modeling: It provides user attributes and movie metadata, which can be integrated with knowledge graphs and semantic tags for enhanced modeling.
- Optimal Size for Experimentation: With its moderate data size, it allows for quick model training and multiple rounds of experimentation, making it an ideal choice for preliminary algorithm validation and performance comparison.

**8. Download Link**

The dataset is available for free download from the GroupLens official website. It is provided in a ZIP compressed format, and once decompressed, the data is in plain text format, making it easy to import into any recommendation system modeling framework for use.

**MovieLens 1M Dataset**

MovieLens 1M (full name: MovieLens 1 Million Ratings Dataset) is a medium-sized recommendation system dataset released by the GroupLens Research Team at the University of Minnesota in 2003. It is one of the most widely used datasets for experimental validation in both academic and industrial settings. Building upon the simple structure of the MovieLens 100K dataset, MovieLens 1M expands the data scale and provides richer user and item feature information, making it suitable for research in various areas, such as deep learning modeling, knowledge graph enhancement, and user profiling.

**1. Dataset Overview**

The MovieLens 1M dataset contains:

- **1,000,209 rating records**
- **6,040 users**
- **3,952 movies**

Compared to MovieLens 100K, the number of users and movies has significantly increased, resulting in a higher data density, making it suitable for training more complex recommendation models and deep learning networks.

**2. File Structure**

The MovieLens 1M dataset is provided in .dat file format and primarily includes the following three core files:

- ratings.dat: Records user ratings for movies.
- users.dat: Contains basic user attribute information.
- movies.dat: Contains basic movie information, including genre, year, and other details.

All files use double colons (::) as field delimiters, making them easy to parse and process.

**3. Rating Data Format**

Each line in the ratings.dat file contains a rating record with the following format:

UserID::MovieID::Rating::Timestamp

Field descriptions:

- UserID: User ID, ranging from 1 to 6040.
- MovieID: Movie ID, ranging from 1 to 3952.
- Rating: User rating, an integer between 1 and 5.
- Timestamp: Rating timestamp, indicating when the rating was given (in seconds, Unix timestamp format).

With over one million total ratings, the dataset has an average of 165 ratings per user, offering a wide rating coverage with moderate sparsity, making it suitable for evaluating model performance on datasets with medium density.

**4. User Information Structure**

User data is stored in the users.dat file, with each line structured as follows:

UserID::Gender::Age::Occupation::Zip-code

Field descriptions:

- Gender: Gender of the user, with M for male and F for female.
- Age: Age group encoded numerically, common encodings include:
  - 1: "Under 18"
  - 18: "18-24"
  - 25: "25-34"
  - 35: "35-44"
  - 45: "45-49"
  - 50: "50-55"
  - 56: "56+"
- Occupation: Occupation ID (ranging from 0 to 20), which can be mapped to specific occupation names.
- Zip-code: Postal code, which can be used for geographical region analysis.

This file supports user preference modeling based on age, gender, and occupation dimensions, as well as group recommendation studies.

**5. Movie Information Structure**

The movies.dat file contains each line in the following format:

MovieID::Title::Genres

Field descriptions:

- Title: The movie title, including the movie name and its release year, e.g., "Toy Story (1995)".
- Genres: A list of movie genres, separated by the pipe (|) symbol, e.g., "Animation|Children|Comedy".
- The dataset includes approximately 20 different movie genres (e.g., Action, Drama, Romance, Sci-Fi, Thriller, etc.), which can be used to build content vectors or tag matrices.

This metadata provides a solid foundation for content-based recommendation methods, tag-enhanced modeling, and multi-label classification.

**6. Data Statistics and Distribution Features**

- User rating distribution is relatively even, with most users rating between 50 to 400 movies.
- On average, each movie is rated about 270 times, with popular movies having over 2,000 ratings.
- Rating distribution is concentrated between 3 and 5, reflecting a user preference tendency and a neutral rating bias.
- A clear long-tail distribution is observed, where some less popular movies have few ratings, making it suitable for cold start experiments.
- The gender ratio is approximately 71% male (M) and 29% female (F), with the age distribution primarily concentrated between 18 and 44 years.

**7. Data Application Advantages**

- Suitable for training complex deep recommendation models and neural collaborative filtering models.
- Supports personalized recommendations based on user attributes and item tags, as well as group behavior analysis.
- Can be used for cold start experiments, interest transfer research, and AIGC-enhanced text-based recommendations.
- High data quality with standardized format, making it easy to integrate with recommendation frameworks such as PyTorch, TensorFlow, and LightFM.

**8. Download Link**

The dataset is available for free download via the GroupLens official website at:

https://grouplens.org/datasets/movielens/1m/

After downloading the compressed file (approximately 5MB), you can extract it to obtain three plain text data files, which can be directly imported into your experimental environment.

**MovieLens 10M Dataset**

The MovieLens 10M dataset, released in 2004 by the GroupLens research team at the University of Minnesota, is one of the standard large-scale recommendation system datasets. It contains over 10 million rating records, making it an enhanced version in terms of data size following the MovieLens 100K and 1M datasets. This dataset balances medium scale, complete structure, and high data quality, making it one of the key resources widely used in recommendation system research for deep model training, large-scale recommendation experiments, and high-dimensional feature modeling.

**1. Dataset Scale and Composition**

The MovieLens 10M dataset contains:

- **10,000,054 user rating records**
- **71,567 user-generated movie tags (Tagging)**
- **69,878 registered users**
- **10,681 movies**

Compared to the 1M dataset, this version demonstrates an order of magnitude increase in the number of users, movies, and interaction density, making it more representative of real-world platform user scale and behavioral complexity.

**2. Dataset File Structure**

The MovieLens 10M dataset is provided in .dat format with fields separated by double colons (::). It mainly consists of the following four core files:

- ratings.dat: Contains user movie rating data.
- movies.dat: Provides basic movie information (title, genre).
- tags.dat: Contains the text tags users have assigned to movies (which can be used for sentiment analysis or tag-based recommendations).
- users.dat: This file is no longer provided due to privacy concerns. In the 10M version, user personal attributes are omitted, and only user IDs are retained.

Additionally, MovieLens 10M offers a .zip format version with optional CSV files for easier processing. It can also be used in conjunction with the Netflix Prize dataset and IMDb data.

**3. Core Fields and Format Description**

**Rating Data (ratings.dat)**

Each line format is:

UserID::MovieID::Rating::Timestamp

- UserID: User identifier (1–69,878)
- MovieID: Movie identifier (1–10,681)
- Rating: Rating value (an integer between 1–5)
- Timestamp: Rating timestamp (in Unix timestamp format)

**Movie Information (movies.dat)**

Each line format is:

MovieID::Title::Genres

- Title: Contains the movie name and release year (e.g., "The Matrix (1999)")
- Genres: Multiple movie genres separated by "|" (e.g., "Action|Sci-Fi|Thriller")

**Tagging Information (tags.dat)**

Each line format is:

UserID::MovieID::Tag::Timestamp

- Tag: User-generated keywords, such as “funny,” “dark humor,” “suspense,” etc.
- These tags can be used to build project semantic features, textual tag graphs, and support AIGC (AI-Generated Content) for automatic tag generation training or reinforcement learning.

**4. Data Statistical Features**

- **High User Activity:** Each user has an average of approximately 143 ratings.
- **Concentrated Rating Distribution:** Ratings of 3 or higher account for over 80%, reflecting a tendency towards neutral or positive ratings from users.
- **Wide Genre Coverage:** The dataset includes over 20 mainstream movie genres, supporting multi-label and multi-category modeling.
- **Presence of Niche Movies:** The rating distribution follows a long-tail pattern, enabling effective research on cold-start and long-tail recommendations.
- **Sparse but Diverse Tag Data:** Although each movie has relatively few tags on average, the tags are semantically rich, making the dataset suitable for pre-training and semantic recommendation modeling.

**5. Applications and Research Value**

MovieLens 10M is an important mid-to-large scale dataset for recommendation system research, offering the following advantages for research applications:

- Supports collaborative filtering and deep recommendation models (e.g., NCF, AutoRec, DIN) training.
- Can be used for AIGC-enhanced recommendations, where tag text content can serve as training data for large language models.
- Suitable for building knowledge graph-based recommendation systems, where movie genres, titles, and user-rating relationships can be converted into graph triples.
- Enables the evaluation of recommendation systems' training efficiency and accuracy in large-scale user-item matrices.
- Unstructured tag information is suitable for advanced tasks such as text classification, tag prediction, semantic recommendation, and explainable recommendations.

**6. Download and Usage Instructions**

The MovieLens 10M dataset can be downloaded for free through the official GroupLens platform:

Download Link: https://grouplens.org/datasets/movielens/10m/

The downloaded file is approximately 65MB in size. After extraction, it contains multiple .dat text files. It is recommended to use Python's pandas.read_csv() function or Spark for data parsing and batch processing.
